# Interweaving Ideas and Patchwork Programmes: Nutrition Projects in Colonial Fiji, 1945–60

**DOI:** 10.1017/mdh.2017.2

**Published:** 2017-04

**Authors:** Sarah Clare Hartley

**Affiliations:** Centre for Global Health Histories, Department of History, University of York, York YO10 5DD, UK

**Keywords:** Colonialism, Fiji, History of Nutrition, Maternal and Child Health, Race, South Pacific

## Abstract

The influence of a range of actors is discernible in nutrition projects during the period after the Second World War in the South Pacific. Influences include: international trends in nutritional science, changing ideas within the British establishment about state responsibility for the welfare of its citizens and the responsibility of the British Empire for its subjects; the mixture of outside scrutiny and support for projects from post-war international and multi-governmental organisations, such as the South Pacific Commission. Nutrition research and projects conducted in Fiji for the colonial South Pacific Health Service and the colonial government also sought to address territory-specific socio-political issues, especially Fiji’s complex ethnic poli,tics. This study examines the subtle ways in which nutrition studies and policies reflected and reinforced these wider socio-political trends. It suggests that historians should approach health research and policy as a patchwork of territorial, international, and regional ideas and priorities, rather than looking for a single causality.

## 1 Introduction

In 1937 the governor of Fiji reported to the British Colonial Office that the islands were a fertile paradise with ‘no shortage of food amongst any section of the population. There is no poverty, such as is known in more highly developed communities’.^1^ While this claim was too bold, it is true that Fiji was not plagued in this period by starvation or prolonged famine on the scale seen in other British territories. During the Second World War, Fiji, unlike many territories in the Pacific, escaped the ravages of frontline warfare and was insulated from the worst food shortages by its agricultural subsistence economy.^2^ Although pronounced deficiency diseases such as kwashiorkor, pellagra and beriberi existed in Fiji in the immediate post-war era, visiting experts and members of the territory’s medical service in the late 1940s and early 1950s agreed that cases were rare, occurring in children as a result of domestic neglect or tragedy – such as the sudden death or incapacity of a parent – or due to natural disasters such as unusually violent hurricanes.^3^ Nevertheless, the colonial administration and the colonial medical service were concerned that sub-clinical nutritional deficiencies – nutrition pathologies without external symptoms or signs – were a widespread and undetected source of general morbidity, exacerbating the infant mortality rate in the colony.^4^ This peak in interest was not unique. Within the metropole the experience of war brought about a shift in governments’ attitudes towards the nutritional health of the nation. Successful rationing, which aimed to maintain the health of the nation under siege whilst providing the army of civilians with wholesome food, forced British governments to recognise that the nutritional status of the nation was, to some degree, their concern.^5^ Moreover, international organisations such as the League of Nations Health Division monitored the effects of insufficient food supplies on European populations during the war, measuring increases in morbidity as well as total mortality and infant mortality, thus highlighting the importance of food relief in controlling the spread of infectious disease.^6^ Subsequently, the newly formed World Health Organization (WHO) urged governments to measure the prevalence of deficiency diseases through nutritional status surveys based on clinical examinations, general trends in child growth and infant mortality, and dietary histories and blood tests of sample groups.^7^

The government of Fiji, with colonial and international support, commissioned nutritional surveys to discern the extent of the problem and to make recommendations to tackle it. These targeted the two main ethnic groups in Fiji – Indo-Fijians and Fijians.^8^ Although the medical services had previously helped define ration-scales for labourers and institutions and provided limited nutrition-education through the Child Welfare Organisation, this research demonstrated a new level of governmental and service responsibility for the nutritional status of the whole population. A.A.J. Jansen, Susan Parkinson and A.F.S. Robertson provide a detailed account of this historical development of nutrition policy in Fiji from their perspectives as medical practitioners, but without reflecting on the impact of international and territorial circumstances.^9^ These external factors included international trends in nutritional science, changing ideas within the British establishment about state responsibility for the welfare of its citizens and of the British Empire for the welfare of its subjects, and the mixture of outside scrutiny and support for projects from post-war international organisations (United Nations agencies (UN)) and multi-governmental organisations (the South Pacific Commission (SPC)). With reference to these wider trends, this study examines how nutrition research and projects conducted in Fiji for the colonial South Pacific Health Service (SPHS) and government sought to address territory-specific socio-political issues, especially Fiji’s complex ethnic politics. It focuses on the period 1945–60 when Fiji’s public services – including the health service – were expanded and developed, before attention was redirected to economic and infrastructure development during 1960s decolonisation. Although pre-war health developments in the Pacific Islands have received scholarly attention, the post-war, pre-independence period remains largely unexplored, particularly in regard to the British territories.^10^ This article addresses the gap in the historical study of a significant period of the development of public health services by exploring the case of Fiji, one of the central, larger island groups.

## 2 The Colonial, Regional and International Context of Nutrition Research

Post-war developments in nutrition policy in Fiji had their roots in the immediate pre-war period. The medical service had responded to pressure from the Christian missions and leading figures within the colony – such as Dr Regina Roberts (wife of the American consul) – to include nutrition instruction in child welfare schemes, but the 1936 Colonial Office dispatch sparked a more concerted drive to improve nutrition within the colony.^11^ Nutrition experts from within Britain (including John Boyd Orr who would subsequently become the first director general of the Food and Agriculture Organization), and internationally, through the League of Nations, lobbied the Colonial Office to measure and reduce the extent of undernutrition in the colonies.^12^ The dispatch received by Fiji became part of the consequent inquiry of the Colonial Office, in 1939, into ‘Nutrition in the Colonial Empire’, which exposed the inadequacy of diets in many of the colonies.^13^ Steps towards colonial and internationally monitored government interventions to improve nutrition in the colonies had begun but were about to be rudely interrupted by war.

The Colonial Office used the report ‘Nutrition in the Colonial Empire’ to highlight the need for development in the empire in a memorandum for the Cabinet in 1939, implying that improving nutrition was both a humanitarian and economic concern as it caused ‘preventable ill health and inefficiency’.^14^ However, post-war, when the rise of the welfare state within Britain and nationalist unrest within many colonies encouraged the British government to affirm its interwar commitment to improving welfare in the empire, nutrition policies were not prioritised when it came to financial aid.^15^ During the war the government passed two Colonial Welfare and Development Acts (1940 and 1945), granting the Colonial Office a pot of £120 million per annum for the expansion of welfare services, which colonial governments could apply for to fund social development projects, and a further £1 million for research projects to inform these.^16^ In order to apply for funding, administrations had to draw up ten-year development plans, the aim of which was to demonstrate how projects would help to strengthen the colony’s economy. Plans that focused too heavily on services without fostering agricultural, infrastructural and industrial changes that would create the economic growth needed to make services self-sustaining did not receive money.^17^ Although nutrition was listed under social development schemes, which received 49% of the total funding under the Acts, only 0.5% of social development funding was spent on nutrition.^18^ This figure refers only to the money spent directly on nutrition research or pilot projects and does not include the much larger sums spent on health services and agricultural development projects in which nutritionists were frequently included or consulted. In Fiji, for example, the Colonial Welfare Development Fund provided a significant part of the £245 942 pounds put towards improving the Colonial War Memorial Hospital in Suva. The hospital would swiftly appoint a dietitian to advise the hospital and to lecture student assistant medical practitioners and assistant nurses on the principles of nutrition, as part of this broader expansion of medical services.^19^ Meanwhile, the first major nutrition survey conducted in Fiji, the Pilot Survey on the State of Nutrition of Fijians and Indians in Fiji, January–February 1950, was funded with colonial welfare and development money.^20^ In funding terms this project was identified as ‘nutrition’, but it was hoped the recommendations it made would be useful to the wider development of health services and agriculture.^21^ Nutrition was an integrated part, but not a pillar, of Colonial Office spending on development.

The Colonial Office also expanded its technical and research services, adding specialist advisory bodies and advisers, and expanding its pool of technical experts in fields such as agriculture, engineering, education, town planning and health by around 6000 personnel.^22^ These provided advice both to the Colonial Office and directly to the colonial administrations. They included the nutritionist Benjamin Stanley Platt (1903–69), who had helped draft the report ‘Nutrition in the Colonial Empire’.^23^ Platt had worked for the Colonial Office in the interwar period and continued to take a particular interest in the colonies and ex-colonies.^24^ Among his interests was nutrition in island territories, and he conducted research for the Colonial Office in the West Indies in the late 1940s.^25^ He was a member of the European Nutrition Society (1941) and headed both the new state-funded Medical Research Council’s human nutrition research unit (1944), and the London School of Hygiene and Tropical Medicine’s new department of nutrition (1946).^26^ The provision and consultation of expertise was another, perhaps more significant way, in which the Colonial Office promoted nutrition work in the colonies. Platt has been taken as an example because he was the main metropolitan expert providing nutrition advice to Fiji. His research team at the London School of Hygiene and Tropical Medicine was asked by the Colonial Office for comments on the Pilot Survey on the State of Nutrition of Fijians and Indians in Fiji, to further inform the colonial authorities when they drew up policy.^27^ Moreover, Platt established a direct correspondence with medical personnel in Fiji, receiving regular updates on nutritional developments there, and visiting in 1962.^28^ Colonial governments were not just provided with advice from the distant metropole but also received visiting experts to conduct specific nutrition projects, for example Lucy Wills (1888–1964), who had previously worked for the Indian medical service, was one of three experts employed on the first nutrition survey in Fiji.^29^ Thus the Colonial Office sought to encourage and supervise nutrition work in the post-war era through expert advice.

Funding and advice from distant Whitehall departments and metropolitan scientific institutions were not the only sources urging colonial administrations to tackle nutrition problems. In the era after Second World War, Britain, along with other colonial powers, banded together to form commissions – multi-governmental regional bodies where metropolitan powers discussed common problems of administration and pooled expertise.^30^ The South Pacific Commission (SPC), established in 1947 as a multi-governmental advisory organisation to foster social, economic and health development in the fifteen non-self-governing Pacific Island territories under the administrations of Australia, Britain, France, the Netherlands, New Zealand and the United States of America, was one of these.^31^ It was partially a response to Chapter 11 of the UN Charter which laid out the responsibilities of governments of non-self-governing territories.^32^ The Charter at once provided internationally recognised guidelines for colonial governance, thus legitimising it, whilst also casting a critical eye on administrations that fell short of them. These responsibilities included ensuring the economic, social and educational advancement of the inhabitants of these territories.^33^ Administrations were also expected to

promote constructive measures of development, to encourage research, and to co-operate with one another and, when and where appropriate, with specialized international bodies with a view to the practical achievement of the social, economic, and scientific purposes.^34^

These international agencies would soon include the United Nations Food and Agricultural Organization (FAO) and the World Health Organization (WHO). The FAO Constitution (1945) stated that the nations accepting the constitution would aim to raise, ‘the levels of nutrition and standards of living of the peoples under their respective jurisdictions’.^35^ The WHO Constitution (1946) defined health as ‘a state of physical fitness and mental and social well-being, not only the absence of infirmity and disease’, stated that the enjoyment of health was a right, and that one of the functions of the organisation was to promote the improvement of nutrition and ‘other aspects of environmental hygiene’ as part of its objective to deliver health.^36^ The Colonial Office and administrations were therefore under external pressure to address nutrition, along with other health and welfare issues. Of more concern to colonial governments was the provision in the Charter that they should ‘develop self-government, to take due account of the political aspirations of the peoples, and to assist them in the progressive development of their free political institutions’.^37^ The establishment of the UN Trusteeship Council which sought to monitor progress towards all these ends in ex-League of Nation mandates or Axis territories (including islands in the Pacific under the control of Australia, New Zealand and the USA), seemed to threaten other administering powers with the spectre of UN surveillance, including through the specialised agencies, meaning that their experts, including nutrition experts, might be received in the territories with suspicion.^38^

It appears Britain was supportive of the SPC, first proposed by Australia and New Zealand in their wartime discussions around the Agreement between Australia and New Zealand of 1944, partially because it seemed to offer a means of deflecting anti-colonial scrutiny. Membership would demonstrate a commitment to social, health and economic development without promising political independence. With France, Britain pushed successfully against the New Zealand and Australian Labour governments’ suggestion that political development should be included in the constitution.^39^ Despite reassurance from Australia’s representative that the constitutional conference had, ‘not drafted any agreement which is directed against anyone. We have not been carving up anybody’s territories’, international relations scholar Gregory Fry has demonstrated that internal tensions between those participants who wanted to use the SPC as paternalist justification for the continuation of empire, and those who wished to use it to end imperialism, continued into the 1950s.^40^

The work of the SPC’s individual social, economic and health departments deserves critical attention because it was through these that the organisation sought to justify its continuing existence. These sections tried to demonstrate that pooling experts and funding would address regionally relevant problems regardless of the differing forms of territorial administration.^41^ Nutrition, particularly relating to maternal and child health, was an early priority of the health section as some degree of post-weaning malnutrition was believed to be a problem shared by the South Pacific islands.^42^ The British–New Zealand SPHS warily welcomed the establishment of the health section, perceiving both beneficial and detrimental potentials for overlap between their remits.^43^ Fear that the SPC had supra-national ambitions, and demarcation disputes between colonial staff and visiting SPC experts occasionally got in the way of peaceable co-operation.^44^ Nutrition projects were no exception and when Sheila Malcolm, an Australian SPC nutrition officer, visited Fiji with suggestions for health education material in 1952 she received a frosty reception from the SPHS’s acting inspector general, Dr Maxwell, and nutrition officer, Susan Holmes (1920–2012). According to a letter from a frustrated Malcolm to her headquarters, ‘they feel we are interfering and nothing I can say makes any difference’.^45^ However, Holmes and Malcolm did arrange information sharing between the two organisations, and the SPHS applied for and accepted funding from the SPC for projects of their own design throughout the period, because their scientific interests in infant nutrition research converged.^46^

While nutrition experts working in Fiji frequently cited work published by the FAO and WHO in the 1950s they did not attend UN agencies’ regional seminars and conferences on nutrition or the related areas of nursing and health education until the 1960s. With the exception of a regional SPC/WHO training course in health education held in 1957, and the secondment of an FAO nutrition-economist to Fiji in 1959 to conduct a market survey (a visit which was curtailed due to lack of progress) published reports had most influence on nutritionists working in the territory.^47^ The FAO and WHO formed a joint expert committee on nutrition in 1949, which set priorities for international research. A discernible trend for quantifying the needs of individual components of diet and a focus on single deficiency disorders can be discerned in the committee’s work.^48^ Their main concern was protein deficiency, but they also devoted attention to disorders such a goitre (iodine deficiency) – both of which would become the focus of research in Fiji.^49^ From 1949–50 the FAO/WHO Expert Committee researched kwashiorkor – a protein deficiency disorder defined by Dr Cicely Williams (1893–1992) of the British colonial medical service in 1933 – and were key in establishing it as the dominant paradigm by which malnutrition in children was understood for the next two decades.^50^ They sought to demonstrate that it differed from pellagra (caused by vitamin B3 deficiency), and marasmus (emaciation caused by protein-calorie shortage).^51^ WHO and FAO field studies in Africa and Central America suggested an alarming presence of protein-deficiency, especially in children of weaning age and they sought to prevent it with the help of UNICEF.^52^ They initially favoured providing skim-milk through supplementary feeding of children based on similar projects in Europe after the war, and because the USA’s milk surplus provided a ready supply with which to carry them out.^53^ School-feeding projects were encouraged as a convenient way of providing nutritious food to children and as a means of educating them on what constituted a good diet.^54^ Recognising that milk supplies could not be quickly increased in many parts of the world the FAO/WHO set up a Protein Advisory Group which was to provide expert advice to the WHO, FAO and UNICEF regarding alternative ‘protein-rich food programs’ between 1955 and 1975.^55^ Among the members of the group was Platt, demonstrating that the colonial and international organisations were happy to consult with the same pool of expertise, which could result in setting similar policy agendas, despite their different ideological outlooks.

Research into the diet of post-weaning children was also an early priority for the SPC. Interwar anxiety about depopulation and critique of Pacific mothering practices meant that infant mortality rates were already an established concern in the minds of administrators and medical personnel in the region.^56^ Britain, New Zealand, Australia and America were also all promoting milk for children and nutrition education for mothers as part of their domestic public health campaigns.^57^ Consequently, the SPC sent nutritionist, Sheila Malcolm on a five-year project, starting in 1949, to investigate the diet and nutritional status of peoples across the region. She was tasked with providing general recommendations to policymakers and creating education materials for the various health services.^58^ Malcolm concluded that infants throughout the South Pacific suffered a lag in weight gain between the ages of nine and eighteen months as a result of low protein consumption and poor weaning practices.^59^ In response the Commission funded studies into the amino acid content of Pacific Island foods in order to try and find suitable protein supplements, employing a biochemist and setting up a laboratory to perform quantitative analysis in Nouméa.^60^ In 1960 the SPC public health officer, William Norman-Taylor, used the relevance of poor nutrition to justify the work of the health section in economic terms. He contended that many Pacific Islanders were trapped in a vicious circle of ill-health, low productivity, low income, poor public services and bad nutrition.^61^ He described the toll of poor nutrition as the ‘slaughter of the innocents’, which he blamed on ‘ignorance of mothercraft’.^62^ In linking poor health to poverty and lack of education he argued that tackling its causes would bring about economic development, whilst avoiding direct comment on modes of governance. Links to economic development were a common means of justifying nutrition work in the period, with the FAO and WHO also espousing this view.^63^

The British Colonial Office, SPC and the UN agencies diverged in their attitudes towards colonialism and in their geographical focus (territorial, regional, and international respectively), and their staff not infrequently engaged in demarcation disputes. However, all were interested in nutrition work from an economic and social development perspective, as well as a health one, and protein deficiency in children was also a priority for each. Therefore, the projects in the following case study should not be imagined as stemming from a coherent, centrally planned research agenda, but neither were they the products of discrete echo chambers. The interests and views of the various actors involved both converged and diverged; and the Colonial Office and colonial government wove together expert findings with their own administrative needs in the development of further nutrition research and programmes.

## 3 Colonial Fiji: Government, Land, Population and Racial Politics

In the immediate post-war decades, the Colonial Office and the government of Fiji faced a dilemma described in *The Times* as the ‘Judgment of Solomon’.^64^ To create a self-governing, multi-racial state within the Commonwealth, it was necessary to divide landownership and political and economic power between the Fijian and Indo-Fijian populations. It appeared to pessimists in the colony and the metropole that historical decisions about the economy and system of land rights in Fiji had created an insurmountable incongruity of interests between these ethnic groups that would prevent political development.^65^ The ways in which race relations affected the development of the Fijian constitution, political parties, land rights and economic development has been studied by scholars such as Brij Lal, Robert Norton, Michael Howard and Steven Ratuva, but its impact on health projects, including nutrition research, and the ensuing health policies and services have not been given similar attention.^66^

The government inherited the consequences of a Victorian colonial policy which had separated Fijians from ‘individualist’ economic activity and excluded Indo-Fijians from landownership. Fijians were governed by a system reminiscent of indirect rule that placed them under the authority of a hierarchy of chiefly officials and administrators, answerable directly to the governor.^67^ A Fijian Secretary for Fijian Affairs protected and administrated Fijian communal landownership, and the alienation of land to any party besides the British Crown was illegal.^68^ The Fijian Chiefs had a close relationship with the European governors and these legal protections were regarded as the ‘Ark of the Covenant’; any apparent challenge to them was likely to strain their accord.^69^ In 1960 Fiji’s land was owned, in descending order of acreage, by: Fijian communities, private (mostly European) landowners, the British Crown, and Fijian individuals.^70^ The majority of Fijians primarily undertook communal subsistence farming which gave them a privileged legal position but somewhat insulated them from what colonial observers termed the ‘individualist’ cash economy.

The reverse was true of the Indo-Fijian community. European plantation owners had been prevented by law from employing a substantial Fijian labour force. Consequently the Sydney-based Colonial Sugar Refining Company, which controlled around 100 000 acres of land and all Fiji’s sugar mills, imported approximately 60 000 indentured labourers from India between 1879 and 1916.^71^ After indenture was outlawed in 1920, many Indians stayed as tenant small-holders and grew sugar cane (Fiji’s main export) for the company.^72^ By the 1950s Indo-Fijians had broken into the professional classes, produced a number of university graduates, and come to dominate motor transport, some branches of business, and sugar cultivation.^73^ The Indo-Fijian community had campaigned for and gained representation on the Legislative Council and had formed an embryonic trade union movement.^74^ They were increasingly politically organised and an integral part of the economy. However, they could only access land through leasehold and, as many Indo-Fijians relied on cane farming for their income, this left them in a vulnerable position.^75^

The 1946 census revealed that Indo-Fijians had outnumbered indigenous Fijians for the first time. This was read as an omen of political instability by the colonial authorities in Fiji and Westminster; the European elite and the Fijian Chiefly class feared that the Indo-Fijians would, like the ‘cuckoo in the nest’, dominate them politically.^76^ The Indo-Fijian birthrate was significantly higher and their infant mortality rate lower than those of the Fijian community during the 1940s and 1950s.^77^ Similarly, although the death rate was decreasing for both Indo-Fijians and Fijians, the Indo-Fijian community retained a lower death rate throughout the period.^78^ By 1960 Indo-Fijians accounted for just under 50% of the population, Fijians for 42%, and Europeans for just under 3% – the remainder of the population included Euronesian, Chinese and Rotuman minorities.^79^

The predominance of the Indo-Fijian community was expected to increase and continue indefinitely putting pressure on land availability. Population growth in Fiji as a whole was rapid and land and population were increasingly politicised and racialised issues.^80^ The Fijian community primarily used their land for subsistence farming, and the concurrent urbanisation and need to supply an expanding market of wage earners was causing further problems.^81^ The colonial government and the Colonial Office hoped to reduce the Indo-Fijian birth-rate and increase Fijian fertility and integration into the cash economy, believing this would stabilise the colony.^82^ The governor of Fiji had suggested in 1937 that nutrition programmes could, ‘improve general stamina, ability to resist disease of the native race, with consequent reaction on fertility and ability to compete economically with immigrant races’. He also hoped that nutritional education would reduce infant and maternal mortality rates and would encourage Fijians to raise stock and grow crops for local consumption and export.^83^

The practicalities and prejudices involved in providing medical services to an ethnically diverse population seeped into nutrition research at a moment when Fiji’s medical service, including its nutrition division, was expanding. Cultural differences in diet prevailed between Fijians and Indo-Fijians as a result of de facto segregation. These were reinforced by food availability. Many Fijians lived in rural areas and relied on community gardens, the surrounding bush, and fishing as their primary sources of food, while Indo-Fijians generally lived in more densely populated areas, sometimes owned a small vegetable plot, and relied on Indian stores to supply them with dry goods. Staple foods for rural Fijians included dalo, tapioca and fish (although urban Fijians often bought European staples such as bread, biscuits and tinned meat), while both urban and rural Indo-Fijians ate dhal, roti, rice and vegetables. Indo-Fijian families consumed milk and dairy products, store-bought or provided by a family goat or cow, while Fijians, as a rule, did not. The only ethnic group for which there was an existing quantitative study of diet was of Indo-Fijian labourers. This took the form of a cost of living survey conducted during a wage dispute in 1939, which had shown their diet was below the USA National Research Council Recommendations for physically active men.^84^ There had been less political impetus for a survey of Fijian diets, and the dominance of subsistence farming presented greater methodological challenges. Post-war researchers were therefore pioneers – and their investigation into the effect of dietary differences on health – and their accompanying social commentary carried weight with the colony’s administration.

## 4 Case Study: Dietary Surveys for the South Pacific Health Service

It was in this international, regional and colonial context that Fiji’s medical service expanded in the 1940s and 1950s. This was a new phase in the history of Britain and New Zealand’s co-operation to overcome the challenges to improving health and medical services in Pacific territories posed by limited resources and poor transport and communication links. Fiji’s capital, Suva, was already the home of the Central Medical Authority (1927) for the Western Pacific High Commission, and the Central Medical School (1929) that trained native auxiliary medical practitioners for British and New Zealand territories.^85^ The New Zealand Nursing Division provided British territories’ medical services with nurses.^86^ Wartime discussions over expanding British–New Zealand co-operation culminated with the establishment of the SPHS (1946) to co-ordinate the health programmes of the New Zealand government and of the British Western Pacific High Commission in their territories.^87^ It pooled staff, sharing the Fijian medical service’s headquarters and director general. In line with international trends the SPHS promoted preventive medicine – establishing a nutrition division and training courses for indigenous nursing and dental assistants with a nutrition component.^88^

Nutrition was a priority for the SPHS from its inception. The second meeting of its executive body stated their intention to improve the nutrition education of medical staff and to research diet and nutritional status in the territories.^89^ The foundations of the Nutrition Division were laid in 1947 by a visit to Fiji from Miss M. Abraham of the Department of Health in New Zealand, to review the existing records of island dietary custom and composition of local food stuffs. She set out dietary standards for pre-school and school age children to be used in schools and orphanages.^90^ In 1948 the Service employed its first full-time dietitian, Miss J.L. King, to supervise hospitals and school diets and to lecture at the Central Medical School and Nursing Training Centre in Fiji. Her salary was raised the following year in recognition of the importance of her service.^91^ Initial observations by these dieticians, and a medical service doctor, suggested that schoolchildren commonly suffered teeth and skin defects and that ‘some degree of nutritional ill-health is present in a large proportion of all children of all races in Fiji’.^92^ Fijian birth and infant weights were noted to be significantly higher than those of Indo-Fijians although they were unsure whether this resulted from a difference in maternal diet or genetics.^93^ To build on these observations the SPHS commissioned a study into ‘Anaemias and other Morbid conditions of Doubtful Origin in Fiji’, to investigate the sources and prevalence of ‘suboptimal nutritional states’.^94^

This resulted in the Pilot Survey on the State of Nutrition of Fijians and Indians in Fiji, January–February 1950 to investigate the source of anaemias and protein and vitamin deficiencies, and an accompanying dental survey.^95^ The nutrition survey was funded by the British Colonial Welfare and Development Fund, the dental research was supported by the Medical Research Council of New Zealand; and three respected outside experts, two from New Zealand and one from Britain, undertook the work. Dr Muriel Bell (1898–1974) headed the survey. Her impressive curriculum vitae included being the first nutrition officer to the Department of Health in New Zealand, the representative of women and children on the Board of Health, chair of the New Zealand Medical Research Council’s nutrition research committee, and Director of Nutrition Research at Otago Medical School.^96^ Her interests were broad, encompassing everything from goitre, to water fluoridation, to possible links between Māori diets and heart disease; she was also an avid campaigner for safe milk in New Zealand, holding long-standing membership of the Milk Council.^97^ She was assisted by Dr Lucy Wills (1888–1964), a British pioneer in haematology, whose primary interest was the biomedical causes of deficiency diseases. Her work in India on pernicious anaemia in pregnancy had led her to identify what then became known as ‘the Wills’ factor’ – the first step to discovering folic acid – and to conduct early trials of iron supplementation for pregnant women.^98^ Dr George Neville Davies (1921–2010), the head of preventive public health and children’s dentistry at the University of Otago, completed the dental survey. He was at an early stage of his career but had already developed an interest in preventive approaches to dental healthcare in New Zealand’s Pacific Island dependencies.^99^ Each brought their own research interests and prior experiences of public health and nutrition to Fiji. The provision of funding and experts by the metropolitan governments demonstrates that they shared the administration’s interest in nutrition.

Bell and Wills focused on Fijians and Indo-Fijians using nineteen 15- to 30-year-old European women as a control group that was perceived to have a healthy diet, because their serum protein levels were within a ‘normal’ range.^100^ They were compared with 110 Fijians and 106 Indo-Fijians within their age range. No European children or older women were included in the sample, and it is unclear whether any pregnant European women were included, comparison with their non-white peers in these categories was therefore methodologically flawed.^101^ This was consistent with the SPHS’s preconceptions; nutrition pamphlets provided by the SPHS for Europeans arriving in Fiji in the early 1950s assumed that European housewives had healthy diets and simply needed advice on finding substitutes for the fresh foods unobtainable on the islands.^102^ Bell and Wills visited hospitals, antenatal and health clinics, and dispensaries to identify common deficiency disorders. They performed clinical examinations on urban and rural populations and several schools, taking blood samples to measure 1002 people’s haemoglobin levels and 820 people’s serum protein levels.^103^ They concluded that ‘malnutrition is not a serious problem’ – that severe vitamin, calorie and mineral deficiencies were rare – but several aspects of diet could be improved. There was a high incidence of moderate anaemia in pregnant Indo-Fijian women, Indo-Fijian women of child-bearing age and their infants, and in Fijian children aged between five and nine. Both Indo-Fijians and Fijians were found to have higher than normal serum protein levels and to be affected by goitre. Dental defects were common, especially in Indo-Fijian children.^104^ Meanwhile Davies compared the dental health of Fijian children living in urban settlements to those in remote villages.^105^ He found a higher incidence of caries in urban areas but that poor tooth development, especially hypo-calcification of deciduous teeth, was common everywhere.^106^ The experts’ interpretations and recommendations, and the response of the government and the medical service demonstrate the influence of international trends, individuals’ research interests, and the different nutritional status and colonial attitudes towards each community.

Bell, Wills and Davies’ reports are central examples because the SPHS and the government of Fiji adopted many of their recommendations, including commissioning further research studies. Their work was the first extensive clinical survey carried out in Fiji, and the first nutrition-related investigation of both Fijian and Indo-Fijian communities. There were no pre-existing dietary studies of Fijian diets, and only a 1937 cost of living survey of Indo-Fijian consumption. The experts were relying on their own impressions and indirect sources of evidence of dietary patterns to relate to their clinical and biomedical findings such as SPHS personnel reports, teachers, and community leader informants.^107^ Some of their interpretations were challenged by researchers working within the colony and the metropole later in the decade, resulting in a patchwork of recommendations for and by the local government and health service.

## 5 Fijian Nutrition

Bell and Wills were particularly critical of Fijian diets despite also finding evidence of poor nutrition amongst Indo-Fijians. This was because they prioritised protein deficiency as a problem and held a Eurocentric view of what constituted a healthy diet. Bell’s public health experience had been limited to Western countries, namely New Zealand where there was a thriving dairy industry, dairy products were a major component of the diet, and where she had developed a personal devotion to the cause of improving milk safety.^108^ The administration they were reporting to had a more paternalist but sympathetic view of Fijians than Indo-Fijians: colonial administrators may have passed on an implicit bias towards prioritising this community through the information that they supplied; and Fijians may have been more welcoming to the researchers than Indo-Fijians. Bell and Wills advised that ‘the magnificent physique of the adolescent and mature Fijian belies the suspicion that protein is grossly deficient in the Fijian diet’, and that ‘the Fijians are in the main a well-nourished race, in contrast with the Indians, in whom cases of nutritional deficiencies turn up in the hospital wards from time to time’.^109^ However, somewhat contrarily, the researchers concluded that Fijian diets were dangerously low in protein because they did not drink much milk. They argued that there was ‘no doubt’ that a lack of milk in Fijian children’s post-weaning diet was responsible for the ‘disquieting’ Fijian infant mortality rate, whereas Indo-Fijians were preserved by a ‘traditional high regard for milk’.^110^ They concluded this, despite finding little evidence of cirrhosis of the liver, primary carcinoma of the liver or nutritional oedema amongst the Fijian population that might indicate the existence of kwashiorkor.^111^ Despite indications that Indo-Fijians were more likely to be hospitalised for nutritional reasons, Bell and Wills did not suggest further studies into their diet. They suggested that because Indo-Fijians had ‘an abundance of milk and other protein foods available’, their smaller stature must be a ‘racial’ trait because they had all the resources for healthy growth.^112^

Bell and Wills’ emphasis on milk and protein consumption in assessing the quality of each community’s diet, reflects the international nutrition forums’ preoccupation with protein deficiency in the 1950s, as well as Bell’s personal concern in promoting the drinking of milk.^113^ Bell and Wills suggested that clinic vans should sell subsidised milk for Fijian infants as in Jamaica – where Nestlé was collaborating with the government to encourage milk production – demonstrating intra-colonial influences. Although they thought the scheme should target Fijians they warned it would be ‘politically unwise’ if Indo-Fijians were excluded.^114^ They offered the services of the nutrition research department in Otago to propose alternative protein foods but warned sceptically that even dried skim-milk would be better than any substitute.^115^

SPHS experts shared Bell and Wills’ concerns that Fijian weaning practices were responsible for their higher infant mortality rate and their concerns about the weaning phase, but were equally worried about the form in which food was given to infants and toddlers. In their view earlier medical teachings based on the New Zealand Plunket system had caused ‘confusion’ by condemning the Fijian practice of feeding children pre-masticated food and of breastfeeding for an extended period, without suggesting suitable replacement practices.^116^ The first inspector general of the SPHS, Dr J.C.R. Buchanan, criticised Fijians for having ‘a tendency to stuff’ children with carbohydrate, while the service’s nutritionist, Susan Holmes, suggested that the primary cause of malnutrition was ‘poor selection and unsatisfactory use of food’ due to the ‘ignorance’ of Fijian mothers.^117^ Doreen Langley (1920–98), an SPHS nutritionist, complained that Fijian mothers weaned abruptly without having supplemented their child’s diet and that the ‘mother was not considered responsible if her child was ill or failed to gain weight’.^118^ She was also concerned that poor hygiene and lack of sleep were impacting children’s health.^119^ Dr C.H.Gurd, who worked at the Colonial War Memorial Hospital in Suva, compared Indo-Fijian weaning routines favourably to Fijian ones because they fed children a digestible ‘soft type of vegetable diet’, supplemented by protein foods such as milk or eggs. He argued that Indo-Fijians followed better hygiene practices, such as feeding children from individual plates, and lived in more populated regions with better access to medical services. For Gurd, these combined factors explained their lower infant mortality rate.^120^ All these colonial employees believed milk was a desirable addition to the diet, but recognised it was expensive and difficult to obtain and believed properly prepared fish, chicken and eggs were adequate substitutes.^121^ Their local knowledge of food supplies and of traditional feeding practices brought them to slightly different conclusions from Bell and Wills. The priority of the SPHS was to re-educate Fijian mothers into preparing their traditional diet in a form suitable for young children and to maintaining breastfeeding for twelve to eighteen months.^122^

The Colonial Office consulted the nutrition division of The London School of Hygiene and Tropical Medicine on Bell and Wills’ report. Platt’s research assistant, Miss Grant, criticised Bell and Wills for ‘a European prejudice’ in favour of milk. Grant believed the actual daily Indo-Fijian consumption of protein suggested by the 1939 cost of living survey could satisfactorily be obtained with Fijian staples, such as fish and dalo.^123^ This reflected Platt’s own research interests into alternative protein foods including coconut milk, which was plentiful in Fiji.^124^ Grant reasoned that the absence of ‘marks on the survivors’ such as stunting (Fijian children averaged the same height as their peers in London) or unusual levels of liver-disease suggested that the Fijian diet was not bad enough to kill large numbers of infants. In her opinion infection was to blame.^125^

Subsequent policies sought to address the concerns of the SPHS and the London School of Tropical Hygiene and Medicine about weaning diets, food supply and hygiene, and Bell and Wills’ specific concerns about Fijian milk consumption. In 1958 the SPHS produced Infant Feeding Booklets in Hindi and Fijian as part of a broader education campaign to improve weaning practices. The booklets included detailed instructions on food hygiene and preparation, advising parents to mash food, boil liquids and to give their baby an individual cup.^126^ Immunisation and tooth-brushing were encouraged.^127^ They also contained comprehensive information on supplementing breastmilk during weaning. Indo-Fijian parents were advised that milk was the best option but that egg, fish, cheese, chicken and dhal were suitable alternative protein foods for an eighteen-month-old child.^128^ However, the Fijian version of the booklet firmly instructed in majuscule that ‘when he is weaned he must have some cow’s milk or skim milk everyday’ and devoted extra pages to explaining how to buy and prepare three ‘cheap’ daily cups of milk.^129^ Although the booklets stressed the importance of maintaining breastfeeding for twelve months, they were sponsored by Nestlé, and several pages were devoted to the preparation of milk powder.^130^ In the same period the government supported a project distributing skim-milk powder to pregnant women and to poor mothers with six or more children in Suva, maintaining an appearance of equanimity by including Indo-Fijian mothers and infants alongside the project’s Fijian targets.^131^ Concurrently the SPC funded the SPHS to investigate the protein content of island foods and to find local substitutes for milk. This was deemed to be of regional importance because of low levels of dairy farming across the Pacific Islands.^132^

Fiji imported a lot of Western tinned goods and Bell saw an opportunity, as an advocate of the New Zealand dairy industry, to improve Fijian nutrition whilst marketing her nation’s surplus dried milk products. Although they found no difference in the serum protein levels between Fijians and Indo-Fijians, which were both higher than the values accepted as normal, Bell and Wills did observe that a greater percentage of Fijian children suffered skin infections. They hypothesised that because Indo-Fijian children drank milk their skin was ‘better equipped for resisting invading organisms’.^133^ They suggested that the higher rate of anaemia in Fijian children aged six to nine was due to skin infections and, therefore, that low milk intake was indirectly contributing to Fijian anaemia.^134^ They preached that ‘no food other than milk can provide the quantities of calcium and riboflavin that are considered necessary for optimum nutrition and growth’.^135^ Davies was more tentative than Bell and Wills in diagnosing the Fijian population with protein deficiency. Although he concluded that a suboptimal intake of dairy products by Fijian pregnant women was the likeliest cause of hypo-calcification in their children, he also considered whether illness during pregnancy was a cause.^136^ The raised serum protein levels Bell and Wills discovered could have indicated exposure to repeated infections, so it is noteworthy that both reports settled on nutritional deficiency as the primary cause of pathology.^137^ While the nexus between infection and nutritional deficiency was known to be complex, their conclusions fell within an international paradigm that put protein deficiency as the root cause of many ills.

The report suggested that dried skim-milk should be provided to schoolchildren.^138^ This was a common form of food programme advocated by both specialised UN agencies and the foreign aid policy of dairy-producing nations such as the USA. Bell and Wills explicitly advocated the trade as well as health benefits of drinking milk. They suggested that New Zealand-style school milk schemes should ‘train the palate’ of Fijian children into buying milk in adulthood.^139^ This would stimulate the Fijian dairy industry but also provide an opportunity for New Zealand to supply milk to ‘bridge’ the existing ‘hiatus’ between supply and demand. In turn, this might develop trade links by which Fiji could sell bananas to New Zealand.^140^ Fijians and Europeans owned the majority of banana plantations in Fiji, and the government was already attempting to incorporate Fijians into the cash economy by encouraging them to expand banana production for export.^141^ The government supported Bell and Wills’ suggestion, introducing school milk schemes in two Fijian areas, Lomaiviti and Moturiki, and encouraging the bulk import of dried skim-milk to increase market supply.^142^ Also addressing the supply concerns raised by SPHS personnel, the government agricultural department introduced *Mozambique tilapia* (a Malaysian fresh water fish), into rural areas that lacked frequent contact with markets.^143^ This was done with SPC assistance as a test of the viability and usefulness of introducing fingerlings to other Pacific Islands.^144^ The health service and the government, for politico-economic reasons and health concerns, were happy to focus their attempts at improving protein and milk supply on the Fijian population when it concerned schoolchildren and the adult population.

Davies was commissioned to investigate the dental health of Fijians despite the greater prevalence of dental problems in the Indo-Fijian and European communities.^145^ His study compared the incidence of tooth decay and poor tooth formation between children living in urban ‘European’ conditions and those in ‘traditional’ *koros*.^146^ This reflected governmental and health service ambivalence towards both urbanisation and traditional Fijian communal life. While the government was keen to encourage the economic participation of individual Fijians in secondary industries, they had invested politically in the traditional hierarchy and feared the consequences of its breakdown. Davies was building on earlier work by private dentist, H.S. Mount, who had been asked during the war to prepare a Council Paper in preparation for the expansion of the Dental Service.^147^ Mount had advocated that Fijians working in urban jobs be encouraged to live in separate village-style communities under the authority of a chief and that some members of these communities should work exclusively to provide native foods. He argued that the higher incidence of dental decay in the urban Fijian population was because Fijians were ‘unaccustomed’ to a European diet (bread, rice, tea and sugar) and should be ‘weaned’ from flour.^148^ He asserted that ‘dental decay is almost certainly a deficiency disease – no less so than beriberi or scurvy’, but did not suggest that Europeans or Indo-Fijians should change their dietary habits despite finding a much higher incidence of tooth decay among them.^149^ Davies was less extreme, but his report also implied that unfamiliarity and ignorance were responsible for dietary problems in urban areas. He argued that Fijians could ‘adapt themselves socially and mentally’ to town life, but, ‘it would appear they tend to “pick out” the worst aspects of the European dietary rather than the best’.^150^ Despite the fact that most urban Fijians in the study were working or living in government institutions, and Mount’s prior criticism of the low income of Fijians, Davies did not question whether they were being provided with the means to eat the ‘best’ European foods.^151^ Instead he proposed that the government raise the price of European foods to prevent Fijians from being able to afford them.^152^

The government continued to monitor the effects of Fijians eating a ‘pseudo-European’ diet in later studies.^153^ On Bell and Wills recommendation, the SPHS carried out a qualitative and quantitative dietary survey of Fijian diets. Doreen Langley was sent to Naduri at six-monthly intervals over two years to monitor the effect of an agricultural department co-operative farm project on Fijians’ diet. Although the government was keen that Fijians make cash earnings from the farm they were anxious that extra income might encourage a poor store-bought diet. Langley found little change in habits; Fijians in Naduri might eat ‘European’ foods such as tea and biscuits for breakfast, but lunch and dinner were traditional fresh-water shellfish and green vegetables.^154^ Urbanisation was a prominent subject internationally, and the government’s concern for health and social implications of urbanisation converged with those of the FAO and SPC. A consumption and market survey was commissioned by the government with FAO and the SPC assistance, to determine how to supply urban Fijians with traditional foods. However, this was curtailed due to conflict between the FAO technical assistance expert, the SPHS, and the agricultural department.^155^

The reports provide glimpses of Fijian reactions to the researchers, albeit mediated through the researchers’ own perceptions, which can be speculatively related to the colonial context. Bell, Davies and Langley commented on the hospitality and interest shown by Fijian Chiefs and villagers in their work. Davies visited rural districts and was dependent on the Fijian hierarchy to facilitate his research. He praised the Fijian administration for organising his transport and lodgings so that the trip ‘went off without a hitch’.^156^ He enjoyed evening entertainments remarking that, ‘without exception the *Bulis* in charge of *Tikinas* were found to be most accommodating, generous, hospitable and helpful’.^157^ He praised his translators, Fijian assistant medical practitioners and school teachers, for sharing their local knowledge of family medical histories with him.^158^ Bell noted that entire Fijian villages gathered interestedly to watch her and Wills extract blood. She was struck by the respect shown to her by young Fijians, who tended to kneel while having their blood drawn. Bell was also welcomed with entertainment and food, although she complained that this took precious time away from her research.^159^ Langley showed gratitude to the Fijian schoolteacher in Naduri, who helped her conduct her dietary survey by encouraging the children in his class to keep food diaries, but was concerned that cultural generosity meant that better than average food was served on the days when she conducted her survey.^160^ The researchers felt that Fijians were not only culturally magnanimous but actively interested in helping their work. Many rural villages under Fijian administrators would not have had frequent visits from unfamiliar Europeans, beyond the local district officer, health sister, and missionary; welcoming researchers may have been a novelty. Moreover, Davies performed around two hundred emergency procedures while visiting remote villages, presenting a rare opportunity to access dental treatment.^161^ This would not be true of villages in the more densely populated parts of Fiji and the welcome and assistance offered to researchers there suggests a reciprocal trust between Fijian leaders and European officials. Bell and Davies were both New Zealanders, they had not come up through the colonial medical service and, although they were likely to have interacted with Pacific Islanders studying at Otago, Fiji was their first or second research trip to a non-Western country respectively.^162^ Bell travelled with a British researcher at all times, so the Fijian community is unlikely to have received her with more or less trust than her British colleague, but Bell and Davies may have commented on their interactions with the Fijian community because the patterns of communal life appeared more ‘foreign’ to them, and therefore of note, than the urban and agricultural lives of Indo-Fijians.

## 6 Indo-Fijian Nutrition

A hint of Fiji’s ethnic politics was present in Bell and Wills’ recommendations on tackling anaemia among the Indo-Fijian population. The researchers focused on Fijian and Indo-Fijian infants, children and women of childbearing age, as the groups most susceptible to anaemia. They found that only 30% of Indo-Fijian women of childbearing age had 90%–100% haemoglobin levels.^163^ Indo-Fijian infants presented a higher incidence of iron deficiency, suggesting a severe maternal iron deficiency in the mother limiting the transference of iron *in utero* or through breastfeeding.^164^ Bell and Wills ascribed this to the lower average age of marriage and higher number of closely spaced pregnancies in Indo-Fijian than the Fijian communities.^165^ They recommended that pregnant women, especially Indo-Fijian women, receive iron supplements, and that the government should raise the legal age of marriage and introduce family planning campaigns.^166^ Bells and Wills’ explanation was plausible – the levels of anaemia in Indo-Fijian children and unmarried teenage girls were normal – and was supported by the London School of Hygiene and Tropical Medicine.^167^ They excluded diet as a major cause of the incongruity between the two groups, despite Wills’ identification of diet as an exacerbating factor in causing pernicious anaemia of pregnancy in India. The omission of a dietary investigation as a possible cause may be in response to the 1939 cost of living survey which suggested that adult adult Indo-Fijian males had an adequate iron intake.^168^

Conversely, Susan Holmes and Carleen O’Loughlin (d. 1971), a researcher of income and wealth, concluded in a 1954 dietary survey that ‘dietary lack of iron must be a predisposing factor’ for Indo-Fijian anaemia.^169^ They argued that urban Indo-Fijians living on wages of £5 to £10 a week received half the iron from their diet that Langley had recorded in her survey of Fijians. They pointed out that dietary iron shortage would take a greater toll on women trying to meet the extra physiological demands of pregnancy and lactation.^170^ Holmes and O’Loughlin also observed that Indo-Fijian women from the few farming families they interviewed had a healthier appearance than the labourers’ wives, which they attributed to their easy access to green vegetables.^171^

There are several possible causes of the different conclusions of each surveys. Bell and Wills lacked up-to-date information on Indo-Fijian household diets and only had a short time in which to familiarise themselves with the colony. Holmes and O’Loughlin’s research was broader in scope, taking account of income, housing and sanitation as well as diet, but they worked with a smaller demographic group within the Indo-Fijian population. As a large portion of the Indo-Fijian population were tenant farmers it is difficult to tell whether urban labourers constituted a representative sample.^172^ However, the very existence of O’Loughlin and Holmes’ study indicates the government and health service were interested in Indo-Fijians’ as well as Fijians’ diets. The difference between Langley’s more anthropologically orientated Fijian survey and the investigation into income and expenditure of Indo-Fijians reflects the divergent relationships between the two groups and the islands’ economy.

From the 1950s the health service offered iron supplements at maternal and child clinics.^173^ A determined family planning campaign also followed in the next two decades, initially targeting the Indo-Fijian community. This had the support of the Colonial Office, the government of Fiji, the health service, a small number of Indo-Fijian leaders and ultimately several international organisations.^174^ It does not appear that the colonial authorities deliberately neglected Indo-Fijians, and their particular deficiency disorders. However, they demonstrated a bias towards enacting recommendations compatible with achieving the socio-political end of reducing Indo-Fijian population growth rather than specifically addressing dietary concerns.

Another dietary deficiency prevalent in the Indo-Fijian community, but also present among Fijians, was goitre. Bell and Wills found that nearly 80% of Indo-Fijian and 30% of Fijian women and girls in their sample were affected.^175^ They worried that there was no common article of diet between the two communities that iodine could be added to, although they suggested the iodisation of salt, a step that had been taken in several countries in the wider Western Pacific and South and East Asian regions and was being studied by the WHO and FAO expert committee.^176^ M.K. Chandulal, from the University of Otago, did a follow-up survey as a dissertation in 1956, demonstrating similar findings in terms of racial distribution of the deficiency in the district of Sigatoka.^177^ Chandulal was able to draw on Langley, and Holmes and O’Laughlin’s findings, identifying that the Fijian diet appeared to supply the minimum dietary requirement of iodine but that of the Indo-Fijians did not.^178^ He noted that a larger percentage of the Indo-Fijians of Sigatoka were ‘prosperous’ than Indo-Fijian urban wage-labourers and concluded that the higher incidence of the deficiency amongst Indo-Fijians might stem from iodine shortages in traditional foods rather than economic necessity.^179^ He suggested that Indo-Fijian women were susceptible because they had more children and undertook more physical labour than their Fijian peers.^180^ The government response to goitre was to try to encourage the bulk importation of iodised salt: an unsuccessful scheme because the extra expense of iodised salt was unpopular with merchants and consumers alike.^181^ His research demonstrates that Indo-Fijian diets were perhaps deficient in several ways, which throws into question whether higher rates of anaemia were caused by higher fertility rates alone.

Only Holmes and O’Laughlin commented on the reaction of Indo-Fijians to their work. They were happy with the assistance of Indo-Fijian teachers in conducting their interviews and believed that they had been provided with a fairly accurate account of expenditure on food and household items by interviewees.^182^ However, they were suspicious that the total income of many homes was higher than the figure given, as weekly expenditure exceeded stated income. They concluded that they had only been told the regular wages of the chief earner but that income from other adult males, overtime, and odd-job income had been omitted.^183^ They suspected that the interviewees were being ‘reticent’ about details such as expenditure on alcohol and quantity of debt.^184^ It would be unsurprising if this were the case, as previous research into urban Indo-Fijian labourers’ living standards had been in relation to wage disputes.^185^ Indo-Fijians might have worried that unguarded explanations of their finances given to even a sympathetic local European like Holmes, would result in the information being used against them in future arbitrations. They were accustomed to Europeans having direct authority to interfere in their lives as landlords, employers and bureaucrats and it was no secret that the European representatives in the Legislative Council were wary of giving Indo-Fijians political power. The mistrust between the two communities may have filtered into the interaction between interviewees and researchers. While Holmes and O’Laughlin’s research provides only limited details in this regard, it is an important reminder that subjects themselves have some control over the amount and type of knowledge available to researchers, and that the impetus for their behaviour may be due to politics as much as individual personalities.

## 7 Conclusion

This study has examined the impact of international, regional and colonial influences on nutrition programmes in Fiji in the immediate post-war period, demonstrating that these were distilled through the filter of the colonial administration’s broader social, political and economic concerns. The SPHS personnel approached nutritional research and activity with enthusiasm in their attempts to expand the health service in the colony. However, the trigger that set the government in motion came from the Colonial Office, spurred on by the League of Nations and British researchers such as John Boyd Orr. The UN applied further pressure on the colonial authorities to sustain the momentum in developing the colonies. The FAO, the WHO, the SPC and the London School of Hygiene and Tropical Medicine lobbied to ensure that nutrition, and specifically deficiency diseases such as protein deficiencies in children, remained on the colonial agenda. Meanwhile the government grappled with a growing land and population crisis aggravated by the ethnic divisions that dominated the colony’s politics, whilst also trying to improve services, including the health service.

The causes and consequences of nutritional status were complex and far reaching, encompassing agricultural policy, educational policy, market control and domestic life. This made improving nutrition a complex challenge for governments, but it also made it an attractive focus for health policy because of the potential to integrate improving nutrition into other social and economic projects. This socio-political context influenced developments in Fiji and this study adds important context to previous histories of nutrition policy developments there.^186^ Researchers for the SPHS, both resident and visiting, worked in this complex web and were also influenced by personal interest and expertise. The resulting research, recommendations and policies were knitted from a mixed wool of predispositions. Post-war colonial preference for the Fijian community, ambivalence over the desirability of integrating Fijians into the cash economy, concern over their higher infant mortality rate, and a long-standing preconception that Fijian women needed to be taught how to be better mothers, imbued research into their nutritional status and diet. The government and health service worked hard to enact the recommendations of researchers for improving the nutrition of this community, through further research, milk programmes and education campaigns. Their paternalist but determined desire to improve Fijian health entwined readily with the international concern about protein deficiency in poor regions, regional and international interest in finding and creating cheap protein foods, and nations marketing their surplus milk for export.

Colonial anxiety about the increasing size and power of the Indo-Fijian community, mixed with a grudging respect for their greater similarity to Europeans in terms of diet, parenting and economic activity, bled into the government response to tackling deficiency diseases affecting them. They were also informed by the international dominance of deficiency diseases as a paradigm for understanding suboptimal nutritional status. This most likely predisposed researchers to treat anaemia, goitre and small stature within the Indo-Fijian community as individual phenomena rather than consequences of a generally poor diet. For the government, individual deficiencies were easier to address politically: for example, iodisation of salt promised a quick fix to the goitre problem. It could also suit other European and Fijian interests because if anaemia was caused by a higher birth-rate then it was a justification to introduce birth-control. Mono-causation also avoided difficult questions, including whether Indo-Fijian labourers were being paid enough, or whether tenants were given enough land and resources to grow their own food as well as cash crops.

The ethnic politics of Fiji has received attention from political and economic historians, but this study indicates the need to investigate the role health studies and programmes had in also reflecting and reinforcing wider socio-political trends in the colony and its impact on the development of services.^187^ More broadly, it suggests that scholars of health research and policy from a historical perspective, rather than looking for straightforward causality, should approach it as a patchwork of territorial, international, and regional ideas and priorities.

## Footnotes

1.
Economic Advisory Council Committee on Nutrition in the Colonial Empire, copy of Despatch no. 43, 26 February 1937, with enclosure, from the Governor of Fiji, Interim Report on Nutrition in Fiji, London School of Hygiene and Tropical Medicine (henceforth LSHTM), Nutrition/17/05/02/01.

2.
H.C. Luke, Governor of Fiji to the Secretary of State for the Colonies, 19 August 1940, LSHTM, Nutrition/17/05/02/01; Robert G. Bowman, ‘Army Farms and Agricultural Development in the Southwest Pacific’, *Geographical Review*, 36, 3 (1946), 423.

3.
South Pacific Health Service (henceforth SPHS), Inspector-General’s Report 1st January 1946 to 30th September 1947, LSHTM, Nutrition 17/05/01/01; S.G. Ross, ‘Pellagra in Fiji’, *American Journal of Tropical Medicine*, 30, 6 (November, 1950), 921–8; Muriel Bell and Lucy Wills, Report on Pilot Survey on State of Nutrition of Fijians and Indians in Fiji, January–February, 1950, The National Archives, Kew (henceforth TNA), CO859/232/6; P.E.C. Manson-Bahr, ‘Fijian Kwashiorkor’, *Documenta de Medicina Geographica et Tropica*, 4 (1952), 97–106.

4.
Paper prepared for 10th Pacific Sciences Congress, ‘The Development in Nutrition Activities in Fiji 1920–60’, LSHTM, Nutrition 17/05/02/05.

5.

Lizzie Collingham , The Taste of War: World War Two and the Battle for Food (London: Allen Lane, 2011), 397–401.

6.

Yves M. Biraud , ‘Health in Europe: A Survey of the Epidemic and Nutritional Situation’, Bulletin Health Organisation (League of Nations), 10, 4 (1943), 557–699; Barona Vilar and Josep Lluis, *The Problem of Nutrition: Experimental Science, Public Health, and Economy in Europe, 1914–45* (Brussels: Lang, 2010), 119–25.

7.
World Health Organization Technical Report Series No. 16, Joint FAO/WHO Expert Committee: Report on the First Session, Geneva, June 1950, 45–58.

8.
The term ‘Indo-Fijian’ denotes people of Indian descent living in Fiji. During the Colonial period officials referred to this group variously as ‘Indians’ or ‘Fiji Indians’ or ‘Indian Fijians’. Due to continuing racial divides in Fiji, there remains political controversy over the term. ‘Indo-Fijian’ is used by academics Brij Lal and Adrian Mayer and has been selected because it clearly differentiates settled Fiji-born people of Indian descent from Indian-born migrants. The term ‘Fijian’ refers to iTaukei as it was officially, and is still commonly, the English term for the indigenous people of Fiji. ‘European’ will describe Caucasians including Australians, North Americans and New Zealanders, as this was the official term used in the Colonial period.

9.
A.A.J. Jansen, Susan Parkinson and A.F.S. Robertson, *Food and Nutrition in Fiji: A Historical Review*, Vol. 1 (Suva: Department of Nutrition and Dietetics, Fiji School of Medicine and the Institute of Pacific Studies of the University of the South Pacific, 1990).

10.

Alexander Cameron-Smith , ‘Australian Imperialism and International Health in the Pacific Islands’, Australian Historical Studies, 1, 41 (2010), 57–74; Alexandra Widmer, ‘Native Medical Practitioners, Temporality, and Nascent Biomedical Citizenship in the New Hebrides’, *Political and Legal Anthropology Review*, 33 (2010), 57–80; Margaret Jolly, ‘Other Mothers: Maternal “Insouciance” and the Depopulation Debate in Fiji and Vanuatu, 1890–1930’, *Maternities and Modernities: Colonial and Postcolonial Experiences in Asia and the Pacific* (Cambridge: Cambridge University Press, 1998), 177–212.

11.

Great Britain, Nutrition Policy in the Colonial Empire, Despatch from the Secretary of State for the Colonies 18 April 1936(London: H.M.S.O, 1936); Jansen, Parkinson and Robertson *op. cit.* (note 9), 399–406.

12.

J.L. Gilks and John Boyd Orr , ‘The Nutritional Condition of the East African Native’, Lancet, 12 (1927), 560–563; James Vernon, *Hunger: A Modern History* (Cambridge, MA: Harvard University Press, 2007), 81–117; Paper prepared for 10th Pacific Sciences Congress, *op. cit.* (note 4); Jansen, Parkinson and Robertson *op. cit.* (note 9), 399–406; Iris Borowy, ‘International Social Medicine between the Wars: Positioning a Volatile Concept’, *Hygiea Internationalis* 6, 2 (2007), 24; Iris Borowy, *Coming to Terms with World Health: The League of Nations Health Organisation 1921–46* (Brussels: Lang, 2009), 387–8.

13.

Great Britain, Economic Advisory Council Committee on Nutrition in the Colonial Empire, First report: Part I, Nutrition in the Colonial Empire, 1938–9(Cambridge: ProQuest LLC, 2007), http://gateway.proquest.com/openurl?url_ver=Z39.88-2004&res_dat=xri:hcpp&rft_dat=xri:hcpp:rec:1938-036300.

14.
Colonial Office Memorandum quoted in Michael Ashley Havinden and David Meredith, *Colonialism and Development: Britain and its Tropical Colonies, 1850–1960* (London: Routledge, 2002), 202.

15.
Collingham, *op. cit.* (note 5), 397–401; Joseph Hodge, Gerald Hödl and Martina Kopf (eds), *Developing Africa: Concepts and Practices in Twentieth-Century Colonialism* (Manchester: Manchester University Press, 2014), 14; Havinden and Meredith, *ibid.*, 201–2.

16.
‘Colonial Development and Welfare Bill’, Nature, 155(24 March 1945), 359.

17.
Havinden and Meredith, *op. cit.* (note 14), 252–5.

18.

*Ibid*., 256.

19.
South Pacific Board of Health, ‘Minutes of the Meeting held at Suva, Fiji on the 13th, 14th and 15th June 1949’ (Fiji: Government Press), LSHTM, Nutrition 17/05/01/01, 4; ‘Millions for Fiji, Plans for Development and Social Services’, *Pacific Islands Monthly*, November (1949), 31; ‘Fiji Progress, Governor Outlines Impressive Programme’, *Pacific Islands Monthly*, November (1946), 8.

20.
Bell and Wills, *op. cit.* (note 3), 29.

21.

*Ibid*., 26–9.

22.
Hodge, Hödl and Kopf (eds.), *op. cit.* (note 15), 15.

23.
Great Britain, *op. cit.* (note 11), 186–201.

24.

Toyin Falola and Christian Jennings , Sources and Methods in African History: Spoken, Written, Unearthed (Rochester, NY: University of Rochester Press, 2003), 335–346.

25.

B.S. Platt , Nutrition in the British West Indies (London: H.M. Stationery Office, 1946).

26.
‘Obituary: Professor B.S. Platt’, *Nature*, 223 (1969), 873–4; Colonel W. Elliot, ‘Nutrition in Colonial Territories’, *Proceedings of the Nutrition Society*, 5, 1–2 (1946), 1–43.10.1079/pns1946006620292691

27.
M.W. Grant to G.H. Clark, 11 February 1952, TNA, CO1023/238.

28.
C.H. Gurd, Physician Specialist, Colonial War Memorial Hospital, Suva, to Professor B.S. Platt, 2 March 1962, Nutrition/17/05/02/02; ‘Report of Visit to Five Fijian villages, an Agricultural Research Station and a Rural Market, by Professor B.S. Platt, L.P. Smith (Supervising Dietitian), District Nurse Siteri from Nausori, Assistant Medical Officer Inoke Qarau from Nausori’, 22 January 1962, LSHTM, Nutrition/17/05/02/03.

29.

Daphne A. Roe , ‘Lucy Wills (1888–1964) A Biographical Sketch’, The Journal of Nutrition, 108, 9 (1978), 1379.35560610.1093/jn/108.9.1377

30.

*Hansard Commons Debate*, 13 July 1943, vol. 391 cc47–151.

31.
Australian Treaty Series 1944 No. 2, Department of Foreign Affairs and Trade Canberra, Australian–New Zealand Agreement 1944 (Canberra, 21 January 1944), clauses 28–30; W.D. Forsyth, ‘The South Pacific Commission’, *Far Eastern Survey*, 18, 5 (9 March 1949), 56–8.

32.
Charter of the United Nations (26 June 1945) Chapter XI; Australian Treaty Series 1944 No. 2, *ibid.*, clauses 28–30; Forsyth, *ibid.*, 56–8; Ernst B. Haas, ‘The Attempt to Terminate Colonialism: Acceptance of the United Nations Trusteeship System’, *International Organization*, 7, 1 (1953), 1–21.

33.
Charter of the United Nations, Chapter XI, Article 73, a.

34.

*Ibid*., Article 73, d.

35.
Constitution of the United Nations Food and Agriculture Organization (Quebec, 16 October 1945), Preamble, 1.

36.
Constitution of the United Nations World Health Organization (New York, 22 July 1946), 2–3.

37.
Charter of the United Nations, Chapter XI, Article 73, b.

38.
Charter of the United Nations, Chapter XIII, Articles 86-91; ‘Trusteeship Council’, United Nations (accessed 21 November 2016), http://www.un.org/en/sections/about-un/trusteeship-council/index.html; United Kingdom High Commissioner in New Zealand, Wellington Conference, 11.30 am, 7 November 1944 and Agreement between Australia and New Zealand signed at Canberra 21 January 1944, 1943/44, TNA, DO114/118; Huntington Gilchrist, ‘V. Colonial Questions at the San Francisco Conference’, *American Political Science Review*, 39, 5 (1945): 982–92; Alexander Hugh McDonald, *Trusteeship in the Pacific* (Sydney: Angus and Robertson, 1949), 20–4; Haas, *op. cit.* (note 32), 1–21.

39.
Australian Treaty Series 1944 No. 2, *op. cit* (note 31), clause 31; ‘Six Nations Pledged to New Deal in South Pacific’, *Advocate* (Burnie, Tas: 7 February 1947), 5, http://nla.gov.au/nla.news-article68987905 (accessed 17 June 2015); ‘Six Nations Meet to Plan Advancement of South Pacific Peoples: South Seas Agreement Drawn up and Signed’, *Advocate* (Burnie, Tas: 7 February 1947), 5, http://nla.gov.au/nla.news-article68987905 (accessed 17 June 2015).

40.
Dr Herbert Evatt quoted in ‘Six Nations Pledged to New Deal in South Pacific’, *Advocate* (Burnie, Tas: 7 February 1947), 5, http://nla.gov.au/nla.news-article68987905p (accessed 17 June 2015); Gregory Fry, ‘The South Pacific “Experiment”: Reflections on the Origins of Regional Identity’, *The Journal of Pacific History*, 32, 2 (1997), 183–4.

41.
‘Six Nations Pledged to New Deal in South Pacific’, *Advocate*, *op. cit.* (note 39), 5; ‘Great Gathering In Suva For Talks On The Pacific’, *The Sydney Morning Herald*, 21 April (1950), 2, http://nla.gov.au/nla.news-article18157419 (accessed 16 June 2015); ‘A Five Year Plan To Develop The South Pacific’, *The Sydney Morning Herald* 23 May (1950), 2, http://nla.gov.au/nla.news-article18169104 (accessed 16 June 2015); ‘Commentary: SPC: Make it of Some Real Use, Or Kill It!’ *Pacific Islands Monthly*, March (1959), 25.

42.
‘South Pacific Commission’, *International Organization*, 2, 3 (1948), 549–50.

43.
‘Minutes of the Meeting held at Suva, Fiji on the 13th, 14th and 15th June 1949’, *op. cit.* (note 19), 5.

44.

Norman J. Padelford , ‘Regional Cooperation in the South Pacific: Twelve Years of the South Pacific Commission’, International Organization, 13, 3 (1959), 382, 390.

45.
Miss Malcolm to Mr Maude and Dr Loison, Health Section of the South Pacific Commission, 25 August 1952, TNA, CO1009/146.

46.
Extract from SPC Progress Report No. 11, 1 April–31 August, 1952, LSHTM, Nutrition 17/05/01/03.

47.
South Pacific Commission, ‘Report on the Follow-up of the SPC/WHO Health Education Training Course, prepared by A.L. Scherzer and E.L. Massal’ (Nouméa: South Pacific Commission, 1958); TNA, CO1009/578.

48.
World Health Organization, June 1950, *op. cit.* (note 7); World Health Organization Technical Report Series No. 44, Joint FAO/WHO Expert Committee: Report on the Second Session (Geneva: WHO, 1951); World Health Organization, *The First Ten Years of the World Health Organization* (Geneva: WHO, 1958), 309–24.

49.
World Health Organization, *The First Ten Years*, *ibid.*, 310–16.

50.
Dr Cicely Williams went on from her career in the colonial medical service to be the first head of the maternal and child health section of the WHO in 1948. The WHO/FAO came under criticism in the 1970s for treating caloric deficiencies as of secondary importance despite mounting evidence that they were a serious problem, starting with Donald S. Maclaren, ‘The Great Protein Fiasco’, *The Lancet*, 304, 7872 (1974): 93–6.

51.

Kenneth John Carpenter , Protein and Energy: A Study of Changing Ideas in Nutrition (Cambridge: Cambridge University Press, 1994), 142–160.

52.

*Ibid*., 149–59.

53.

Jean Ritchie , Learning Better Nutrition: A Second Study of Approaches and Techniques (Rome: FAO, 1967), 74–76.317651

54.

Jean Ritchie , Teaching Better Nutrition: A Study of Approaches and Techniques (Washington: FAO, 1950), 69–70; Marjorie Scott, *School Feeding: Its Contribution to Child Nutrition* (Rome: FAO, 1953).

55.

Richard D. Semba , ‘The Rise and Fall of Protein Malnutrition in Global Health’, Annals of Nutrition and Metabolism, 69, 2 (2016), 79–88.2757654510.1159/000449175PMC5114156

56.
Jolly, *op. cit.* (note 10), 177–212.

57.

Philippa Mein Smith , ‘New Zealand Milk for “Building Britons”’, Medicine and Colonial Identity (London & New York: Routledge, 2003), 79–102; R.D. Apple, ‘A Century of Infant Feeding in the United States and New Zealand’, *Occasional Papers on Medical History Australia*, 6 (1992), 303–10; Glenda Mather, ‘Bringing up Baby: 1950s Maternal and Infant Nutrition Programmes’, *Outskirts Online Journal*, 7 (November 2000),http://www.outskirts.arts.uwa.edu.au/volumes/volume-7/mather.

58.
South Pacific Commission to Brigadier H.J.M. Flaxman, British Resident Commissioner, New Hebrides, 18 January 1951, TNA, CO1009/146.

59.
Memorandum, Lectures and Posters on Nutrition, prepared by Dr E. Massal and Miss S. Malcolm, TNA, CO1009/146; Dr Guy Loison, ‘International Seminar on Health Education’, *South Pacific Commission Bulletin*April (1956), 29–32.

60.
Project No. H2 and H5: Diet and Nutrition, 1950–5, TNA, CO1009/146.

61.

W. Norman-Taylor , ‘The Advance of Public Health’, *South Pacific Commission Bulletin*, October (1960), 66–8.

62.

*Ibid*., 67.

63.
Joint FAO/WHO Expert Committee on Nutrition, ‘Report on the Second Session, Rome, 10–17 April 1951’, 29–33; United Nations, *Formulation and Economic Appraisal of Development Projects: Lectures delivered at the Asian Centre on Agricultural and Allied Projects, Training Institute on Economic Appraisal of Development Projects, Lahore, Pakistan, October–December 1950*, Vol. 2 (Lahore: United Nations Technical Assistance Administration, 1951) 515–51, 569–75.

64.

*The Times* (London: Thursday, 13 June 1957), Issue 53866, 9.

65.
Extract from Policy in Fiji note by Mr Amery, Parliamentary Under-Secretary of State to the Colonies, 8 November 1960, TNA, CO1036/811; Documents 5–8 in Brij V. Lal, *Fiji* (London: TSO, 2006), 132–79.

66.

Michael C. Howard , Fiji: Race and Politics in an Island State (Vancouver: UBC Press, 1991); Brij V. Lal, *Broken Waves a History of the Fiji Islands in the Twentieth Century* (Honolulu: University of Hawaii Press, 1992); Robert Norton, ‘Accommodating Indigenous Privilege: Britain’s Dilemma in Decolonising Fiji’, *The Journal of Pacific History*, 37, 2 (2002), 133–56; Steven Ratuva, ‘Proto-Affirmative Action: Indigenous Fijian Development from Cession to Independence’, *The Politics of Preferential Development: Trans-Global Study of Affirmative Action and Ethnic Conflict in Fiji, Malaysia and South Africa* (Canberra: ANU Press, 2013), 11–29, http://press.anu.edu.au?p=245891.

67.

Brij V. Lal , Historical Dictionary of Fiji (Lanham, MD: Rowman & Littlefield, 2015), xxiii.

68.

*Ibid*., xxxii–xxxv, 154, 185.

69.
Professor O.H.K. Spate ‘Time for Tapa Curtain to be Taken Down’, TNA, CO1036/811; ‘Fiji Talanoa, Vakatawa talks of This and That, Fiji Land and Deed of Cession’, *Pacific Islands Monthly*, April (1959), 49.

70.
3 756 000 acres of Fijian communal land, 447 000 acres of private freehold, 215, 500 acres of Crown protected land and 12 000 acres of Fijian freehold, O.H.K. Spate, *The Fijian People: Economic Prospects and Problems, a Report* (Suva, 1959), para 29.

71.
Lal, *op. cit.* (note 67), 63.

72.

*Ibid*., 9.

73.

A.C. Cato , ‘Fijians and Indo-Fijians: A Culture Contact Problem in the South Pacific’, Oceania, 26 (January 1955), 16–17.

74.
Lal, *op. cit.* (note 66), 86–95.

75.
‘Indian Cry for Land is Fiji’s most Pressing Problem’, *Pacific Islands Monthly*, November (1946), 11; ‘Over Quarter of a Million People now in Fiji’, *Pacific Islands Monthly*, April (1947), 17; ‘Governor warns Fiji Indians of Over-Population Peril: Britain will maintain Pledges given to Fijians’, *Pacific Islands Monthly*, August (1949), 9, 91.

76.
Fiji, *A Report on the Results of the Census of Population, 1946* (Suva: Fiji, 1947); Colonial Office Notes, 17 December 1952, TNA, CO1023/212; Document 7 in Lal, *op. cit.* (note 65), 23; ‘Growing hold of Indians on Fiji’s Economic Life’, *Pacific Islands Monthly*, September (1949), 59.

77.
Based on birth rate and population from Rajesh Chandra and Jenny Bryant-Tokalau, *Population of Fiji* (Nouméa, New Caledonia: South Pacific Commission, 1990); infant mortality from, *Fiji: Economic Development of the People*, 1951–4, TNA, CO1023/172, 1951; Mrs Ena Compton, ‘Report of Survey into the Need for Family Planning in the Pacific Islands of Fiji, Tonga, Niue & Eastern & Western Samoa, August 1957’, no page numbers, TNA, CO1036/431.

78.

*Annual report on Fiji* (London: TSO, 1948–60).

79.

*Annual report on Fiji* (London: TSO, 1960).

80.
Total population of Fiji grew from 260 468 in 1946 to 401 018 in 1960, *Annual Report on Fiji* (London: TSO, 1948–60).

81.
Spate, *op. cit.* (note 70), para 29; Fiji: Proposed Commission of Enquiry to Investigate Population Problems, 1952–3, TNA, CO1023/212; A Plan of Operation for a Market Study to be done in the Colony of Fiji, 1960, LSHTM, Nutrition 17/05/06.

82.
Fiji: Proposed Commission of Enquiry, *ibid.*; Burn’s Commission, 1960–2, TNA, CO1036/811.

83.
Economic Advisory Council Committee, *op. cit.* (note 1).

84.
Bell and Wills, *op. cit.* (note 3), Appendix 3.

85.

Annie Stuart , ‘Contradictions and Complexities in an Indigenous Medical Service’, The Journal of Pacific History, 41, 2 (2006), 125–143; South Pacific Board of Health, ‘Minutes of the Meeting held at Suva, Fiji on the 25th, 28th and 31st October 1946’ (Fuji: Government Press), LSHTM, Nutrition 17/05/01/01, 1.

86.

*Ibid*.

87.

*Ibid*.; British territories: Fiji, the Gilbert and Ellice Islands Colony, British Solomon Islands protectorate, the New Hebrides and Pitcairn Island and Tonga (protectorate). New Zealand dependencies: Tokelau, the Cook Islands and Western Samoa.

88.
Inspector-General’s Report 1st January 1946 to 30th September 1947, *op. cit.* (note 3), 5–6.

89.
South Pacific Board of Health, ‘Minutes of the Meeting held at Suva, Fiji on the 20th, 21st, 24th and 27th October 1947’ (Fuji: Government Press), LSHTM, Nutrition 17/05/01/01.

90.
Jansen, Parkinson and Robertson, *op. cit.* (note 9), 340–9; Inspector-General’s Report 1st January 1946 to 30th September 1947, *op. cit.* (note 3), 5–6.

91.
‘Minutes of the meeting held at Suva, Fiji on the 13th, 14th and 15th June 1949’, *op. cit.* (note 19), 4.

92.
Inspector-General’s Report 1st January, 1946 to 30th September, 1947, *op. cit.* (note 3), 5–6.

93.

*Ibid*, 5–6; Muriel E. Bell, ‘A Nutrition Officer’s Experiences Visiting the Southwest Pacific Islands’, *Nutrition Reviews*, 14, 2 (1956), 35.10.1111/j.1753-4887.1956.tb01462.x13288943

94.
Inspector-General’s Report 1st January, 1946 to 30th September, 1947, *op. cit.* (note 3), 8.

95.
Bell and Wills, *op. cit.* (note 3); Bell, *op. cit.* (note 93), 33.

96.
Philippa Mein Smith, ‘Bell, Muriel Emma’, *The Dictionary of New Zealand Biography, Te Ara – The Encyclopedia of New Zealand*, 5 June 2013, http://www.TeAra.govt.nz/en/biographies/4b21/bell-muriel-emma/(accessed 28 April 2016).

97.
Smith, *ibid.*; Katherine Keller, *Encyclopedia of Obesity* (Thousand Oaks, CA: Sage, 2008), 68.

98.

H. Bastian , ‘Lucy Wills (1888–1964): The Life and Research of an Adventurous Independent Woman’, The Journal of the Royal College of Physicians of Edinburgh, 38, 1 (2008), 89–91.19069045

99.
Jennifer Smyth and John Davies, ‘The Dentist Who Professed that Prevention was far better than Cure’, *The Sydney Morning Herald*, 26 February (2010), http://www.smh.com.au/comment/obituaries/the-dentist-who-professed-that-prevention-was-far-better-than-cure-20100225-p5sr.html#ixzz477yIUisd(accessed 28 April 2016); G. N. Davies, ‘Pukapuka Research Expedition, 1950–1’, LSHTM, Nutrition 17/05/02/10.

100.
Bell and Wills, *op. cit.* (note 3), 23, Table 2.

101.

*Ibid*., 23, Table 2.

102.
Tropical Food and Nutrition, Quarterly Pamphlet Issues by the SPHS, LSHTM, Nutrition 17/05/01/03.

103.
These figures excluded patients selected by local health sisters for pathological diagnosis. Bell and Wills, *op. cit.* (note 3), 22.

104.
Bell and Wills, *op. cit.* (note 3), 25.

105.
G.N. Davies, ‘A Report on the Dental Status of Native Fijians’, LSHTM, Nutrition/17/05/02/13, 1.

106.
G.N. Davies, ‘A Report on the Dental Status of Native Fijians’, Part Two, 23, 1950, TNA, CO1023/238.

107.
Davies, *op. cit.* (note 105), 1; M.W. Grant to G.H. Clark, 11 February 1952, TNA, CO1023/238.

108.
Smith, *op. cit.* (note 96).

109.
Bell and Wills, *op. cit.* (note 3), 23; Bell, *op. cit.* (note 93), 34.

110.
Bell and Wills, *op. cit.* (note 3), 23.

111.

*Ibid*., 26–7.

112.

*Ibid*., 23.

113.
Joint FAO/WHO Expert Committee on Nutrition: Report on the Second Session, Rome, 10–17 April 1951; Joint FAO/WHO Expert Committee on Nutrition: third report of a meeting held in Fajara, Gambia, from 28 November to 3 December 1952; Smith, *op. cit.* (note 96).

114.
Bell and Wills, *op. cit.* (note 3), 26–7.

115.

*Ibid*., 26.

116.
Jansen, Parkinson and Robertson, *op. cit.* (note 9), 349–50.

117.

J.C.R. Buchanan , A Guide to Pacific Island Dietaries (Wellington (NZ): H.H. Tombs, 1947), 47; Susan Holmes quoted in ‘Protein Malnutrition, Problem and Prevention in South and East Asia’, *South Pacific Commission Bulletin*, October (1953), 17, 23.

118.
Doreen Langley, ‘Report on Dietary Survey, Naduri, Sigatoka, June–July 1952’, no page numbers, LSHTM, Nutrition 17/05/06.

119.

*Ibid*., no page numbers.

120.
C.H. Gurd, Physician Specialist, Colonial War Memorial Hospital to Professor BS Platt, 2 March 1962, LSHTM, Nutrition/17/05/02/02.

121.
Buchanan, *op. cit.* (note 117), 47; similar view expressed by C.H. Gurd, Physician Specialist, Colonial War Memorial Hospital to Professor B.S. Platt, 2 March 1962, LSHTM, Nutrition/17/05/02/02.

122.
SPHS, *English Translation of Fijian ‘Infant Feeding Booklet’*, 1958, LSHTM, Nutrition 17/05/02/14.

123.
M.W. Grant to G.H. Clark, 11 February 1952, TNA, CO1023/238.

124.

Dana Raphael , International Conference on Human Lactation, Breastfeeding and Food Policy in a Hungry World (New York: Academic Press, 1979), 256.

125.
M.W. Grant to G.H. Clark, 11 February 1952, TNA, CO1023/238.

126.
SPHS, *English Translation of Fijian*, *op cit.* (note 122); *English Translation of Hindi ‘Infant Feeding Booklet’*, 1958, LSHTM, Nutrition 17/05/02/14.

127.
SPHS, *English Translation of Fijian*, *op cit.* (note 122), 24; *English Translation of Hindi*, *op. cit.* (note 126), 18–21.

128.
SPHS, *English Translation of Hindi*, *op. cit.* (note 126), 18.

129.
SPHS, *English Translation of Fijian*, *op cit.* (note 122), 21–3.

130.

*Ibid*, 7–8; *English Translation of Hindi*, *op. cit.* (note 126), 8.

131.
Inspector-General’s Report 1956/7 (Suva, Fiji: Government House, 1957), LSHTM, Nutrition 17/05/01/01, 23.

132.
Recommendations on Health Projects H2. & 5: Nutrition including Infant Feeding, RC. 3/34/Rev.1, 25 June 1951, LSHTM, Nutrition 17/05/01/01.

133.
Bell and Wills, *op. cit.* (note 3), 24.

134.

*Ibid*., 23–4.

135.

*Ibid*., 24.

136.
Davies, *op. cit.* (note 106), 8, 22.

137.
Bell and Wills, *op. cit.* (note 3), 25.

138.

*Ibid*., 26–7.

139.

*Ibid*., 27, 29.

140.

*Ibid*., 29.

141.
‘Fiji Maintains Trade with NZ’, *Pacific Islands Monthly*, June (1947), 60; ‘Higher Price for Fiji Bananas in NZ’, *Pacific Islands Monthly*, April (1950), 37; ‘A Brief Lesson in Banana Importing: Simple and Easy He Says! But Has He Tried it?’, *Pacific Islands Monthly*, April (1959), 31; ‘Diseases and Australians seen as a Threat to Islands’ Banana Markets’, *Pacific Islands Monthly*, November (1964), 9.

142.
Introductory Notes for a Symposium concerning Problems of Applied Nutrition in the Pacific Islands (for 10th Pacific Science Congress), 1961, LSHTM, Nutrition/17/05/02/08; Jansen, Parkinson and Robertson, *op. cit.* (note 6), 359.

143.
Jansen, Parkinson and Robertson, *op. cit.* (note 9), 359.

144.

W.J.A. Payne , ‘Sigatoka Agricultural Station: 2’, South Pacific Commission Bulletin, July (1955), 27.

145.
‘Nutrition: Dental Conditions in Fiji’, Council Paper No. 14, Legislative Council Fiji, 1941, 3, TNA, CO/859/67/11; Bell and Wills, *op. cit.* (note 3), 25; Davies, *op. cit.* (note 106), 15.

146.
Davies, *op. cit.* (note 106), 4.

147.
‘Nutrition: Dental Conditions in Fiji’, *op. cit.* (note 145).

148.

*Ibid*., 2–3.

149.

*Ibid*., 3.

150.
Davies, *op. cit.* (note 106), 8–9.

151.
‘Nutrition: Dental Conditions in Fiji’, *op. cit.* (note 145), 2.

152.
Davies, *op. cit.* (note 106), 8–9.

153.
Davies, *op. cit.* (note 106), 23.

154.
Doreen Langley, Report on Dietary Survey, Naduri, Sigatoka, 1952/3, no page numbers, Nutrition 17/05/06.

155.
A Plan of Operation for a Market Study to be done in the Colony of Fiji, LSHTM, Nutrition 17/05/06; South Pacific Commission: Nutrition Programmes, 1955–62, TNA, CO1009/578.

156.
Davies, *op. cit.* (note 105), 5–7.

157.

*Ibid*., 5–7.

158.

*Ibid*., 5–7.

159.
Bell, *op. cit.* (note 93), 33.

160.
Doreen Langley, ‘Report on Second Dietary Survey, Naduri, Sigatoka, January, 1953’, no page numbers, LSHTM, Nutrition 17/05/06.

161.
Davies, *op. cit.* (note 105), 14.

162.
Smith, *op. cit.* (note 96); Smyth and Davies, *op. cit.* (note 99).

163.
Bell and Wills, *op. cit.* (note 3), 22.

164.

*Ibid*., 22.

165.

*Ibid*., 22.

166.

*Ibid*., 27.

167.
M.W. Grant to G.H. Clark, 11 February 1952, TNA, CO1023/238.

168.
Bell and Wills, *op. cit.* (note 3), 23, Appendix 3. Roe, *op. cit.* (note 29), 1379–83.

169.
Carleen O’Loughlin and Susan Holmes ‘A Survey of Economic and Nutritional Conditions in Indian Households (in Fiji)’ (May 1954), 20, LSHTM, Nutrition/17/05/02/11.

170.

*Ibid*., 20.

171.
O’Loughlin and Holmes, *op. cit.* (note 169), 20.

172.

*Ibid*., 20.

173.
Jimaima T. Schultz and Penina T. Vatuwaqa, ‘Impact of Fortified Flour in Childbearing Age Women in Fiji 2010 report’ (Suva, Fiji: National Food and Nutrition Centre, March 2012), 1.

174.
Part of thesis in progress by the author titled ‘Feeding Health: The Politics of Health Education and Family Planning in Fiji 1945–74’, University of York.

175.
Bell and Wills, *op. cit.* (note 3), 25.

176.
Joint FAO/WHO Expert Committee on Nutrition, Report on the First Session, Geneva 24–8 October 1949 (Geneva: WHO, 1950), 13; World Health Organization Technical Report Series No. 97. Joint FAO/WHO Expert Committee: Fourth Report (Geneva: WHO, 1955), 39–41.

177.
M.K. Chandulal, ‘Goitre in Sigatoka, Fiji’, Department of Preventive and Social Medicine, Dissertation, Medical school, University of Otago, 1956, no page numbers, LSHTM, Nutrition/17/05/02/03.

178.

*Ibid*.

179.

*Ibid*.

180.

*Ibid*.

181.
Inspector-General’s Report 1956/7, *op. cit.* (note 131), 22; Introductory Notes for a Symposium, *op. cit.* (note 142).

182.
O’Loughlin and Holmes, *op. cit.* (note 169), 1, 3.

183.

*Ibid*., 2.

184.

*Ibid*., 7–8.

185.
Bell and Wills, *op. cit*. (note 3), Appendix 3.

186.
Jansen, Parkinson and Robertson, *op. cit.* (note 9).

187.
Lal, *op. cit.* (note 66); Norton, *op. cit* (note 66), 133–56; Howard, *op. cit.* (note 66); Ratuva, *op. cit.* (note 66).

